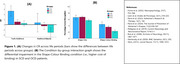# Cognitive reserve and memory markers in the dementia continuum

**DOI:** 10.1002/alz70857_106715

**Published:** 2025-12-25

**Authors:** Mario A A Parra, Daniela Thumala, Patricia Lillo, Rodrigo Saguez, Andrea Slachevsky

**Affiliations:** ^1^ University of Strathcylde, Glasgow, Glasgow, United Kingdom; ^2^ Geroscience Center for Brain Health and Metabolism (GERO), Santiago, Chile; ^3^ Laboratory of Neuropsychology and Clinical Neuroscience (LANNEC), Physiopathology Program‐ICBM, East Neurologic and Neurosciences Departments, Faculty of Medicine, University of Chile, Santiago, Chile; ^4^ Department of Psychology, University of Chile, Santiago, Chile; ^5^ Geroscience Center for Brain Health and Metabolism (GERO), Santiago, Metropolitana, Chile; ^6^ Department of Neurology South, Faculty of Medicine, University of Chile, Santiago, Chile; ^7^ Neuropsychology and Clinical Neuroscience Laboratory (LANNEC), Physiopathology Department – Institute of Biomedical Sciences (ICBM), Neuroscience and East Neuroscience Departments, Faculty of Medicine, Universidad de Chile, Santiago, Chile, Santiago, Chile; ^8^ Memory and Neuropsychiatric Clinic (CMYN), Neurology Service, Hospital del Salvador and Faculty of Medicine, University of Chile, Santiago, Chile; ^9^ Memory and Neuropsychiatric Center (CMYN) Neurology Department, Hospital del Salvador and Faculty of Medicine, University of Chile, Santiago, Region Metropolitana, Chile; ^10^ Neuropsychology and Clinical Neuroscience Laboratory (LANNEC), Physiopathology Department ‐ ICBM, Neuroscience and East Neuroscience Departments, Faculty of Medicine, Universidad de Chile, Santiago, Chile

## Abstract

**Background:**

Cognitive reserve can render neuropsychological assessments for dementia unreliable (Pettigrew & Soldan, 2019). It is commonly measured using proxy variables such as educational attainment, intellectual functioning or social engagement (Stern, 2012). The Visual Short‐Term Memory Binding Test (VSTMBT) and the Free and Cued Selective Reminding Test (FCSRT) can inform on the dementia continuum (Forno et al., 2022; Parra et al., 2022). However, the FSCRT (Roe et al., 2008) but not the VSTMBT (Parra et al., 2024) has proved sensitive to CR. We investigated if a more reliable measure of CR (León et al., 2016) could dissociate across these tests.

**Methods:**

We involved 414 participants (42 Healthy Controls ‐ CTR, 213 with Subjective Cognitive Decline – SCD, and 159 with major Objective Cognitive Decline ‐ OCD) from the GERO cohort (Slachevsky et al., 2020). We also used the VSTMBT, FCSRT, and the new CR Scale. GLM explored changes in CR across three life periods (youth, adulthood, and maturity), binding abilities, and groups. Adjusted stepwise regression models explored the predictive value of Group and CR changes (youth‐adult, adult‐maturity) on memory binding.

**Results:**

The neuropsychological assessment revealed the pattern ((CTR ≥ SCD) > OCD, all *p* < 0.05). CR decreased across life periods, with SCD and OCD patients displaying the most significant drop (CR Change *p* <0.001, Group *p* <0.001, Interaction *p* = 0.221) (Figure 1A). Specific binding deficits were confirmed in SCD and OCD [*F*(2,346)=3.38, *p* =  0.035, η^2^ = 0.02, β=63%] (Figure 1B). CR did not predict the cost of binding (VSTMBT), with only Group retained (R^2^ = 1.8%, *F* = 5.99, *p* = 0.015). However, Immediate Free Recall (FCSRT) was best predicted by a model that retained CR, MoCA, Education and Age (R^2^ = 30.1%, *F* = 25.83, *p* <0.001).

**Conclusions:**

Binding functions assessed by the VSTMBT and the FCSRT are differentially affected by CR. VSTMB is a low‐level visual cognition function less influenced by factors underpinning CR. The FCSRT taps into a broader network that involves language, mnemonic, and executive abilities. Future efforts should be directed to exploring versions of these promising tests that can circumvent the influence of CR and thus provide timely and reliable evidence of dementia risk.